# Hemispheric asymmetries in mental disorders: evidence from rodent studies

**DOI:** 10.1007/s00702-023-02610-z

**Published:** 2023-02-26

**Authors:** Annakarina Mundorf, Sebastian Ocklenburg

**Affiliations:** 1 Institute for Systems Medicine and Department of Human Medicine, MSH Medical School Hamburg, Hamburg, Germany; 2 Department of Psychology, Medical School Hamburg, Hamburg, Germany; 3 ICAN Institute for Cognitive and Affective Neuroscience, Medical School Hamburg, Hamburg, Germany; 4 Institute of Cognitive Neuroscience, Biopsychology, Department of Psychology, Ruhr-University Bochum, Bochum, Germany

**Keywords:** Motor asymmetry, Atypical, Lateralization, Neurodevelopmental, Neurodegenerative

## Abstract

The brain is built with hemispheric asymmetries in structure and function to enable fast neuronal processing. In neuroimaging studies, several mental disorders have been associated with altered or attenuated hemispheric asymmetries. However, the exact mechanism linking asymmetries and disorders is not known. Here, studies in animal models of mental disorders render important insights into the etiology and neuronal alterations associated with both disorders and atypical asymmetry. In this review, the current literature of animal studies in rats and mice focusing on anxiety and fear, anhedonia and despair, addiction or substance misuse, neurodegenerative disorders as well as stress exposure, and atypical hemispheric asymmetries is summarized. Results indicate overall increased right-hemispheric neuronal activity and a left-sided behavioral bias associated with symptoms of anxiety, fear, anhedonia, behavioral despair as well as stress exposure. Addiction behavior is associated with right-sided bias and transgenic models of Alzheimer’s disease indicate an asymmetrical accumulation of fibrillar plaques. Most studies focused on changes in the bilateral amygdala and frontal cortex. Across studies, two crucial factors influencing atypical asymmetries arose independently of the disorder modeled: sex and developmental age. In conclusion, animal models of mental disorders demonstrate atypical hemispheric asymmetries similar to findings in patients. Particularly, increased left-sided behavior and greater right-hemispheric activity were found across models applying stress-based paradigms. However, sex- and age-dependent effects on atypical hemispheric asymmetries are present that require further investigation. Animal models enable the analysis of hemispheric changes on the molecular level which may be most effective to detect early alterations.

## Introduction

Hemispheric asymmetries, that is, differences between the left and the right hemisphere are present on the structural, functional, and molecular level (Mundorf et al. 2021b; Kong et al. 2022; Ocklenburg et al. 2022). In the human brain, structural hemispheric asymmetries are present in the surface area of 91.1% of cortical regions and in the thickness of 76.5% of cortical regions (Kong et al. 2022). Equally high percentages have been observed for subcortical regions (Guadalupe et al. 2017). Similarly, functional asymmetries are present in left–right differences in the processing of language, faces, or emotions in the brain (Corballis 2019; Ocklenburg et al. 2022). This asymmetric organization of the brain holds several benefits for the organism as the hemispheric specialization allows to avert bilateral interference, reduces redundancy, and thus enables a more energy-efficient design and increased multitasking capabilities and action control (Vallortigara and Rogers 2020; Ocklenburg and Mundorf 2022). Atypical hemispheric asymmetries may result in negative consequences for the individual (Ocklenburg and Mundorf 2022).

Fascinatingly, altered or reduced hemispheric asymmetries have been repeatedly associated with several neurodevelopmental, psychiatric, and neurodegenerative disorders (Mundorf and Ocklenburg 2021; Kong et al. 2022). Recently, large analyses spanning thousands of participants have been conducted for some disorders by an ENIGMA Consortium, which revealed atypical structural asymmetries in patients diagnosed with autism spectrum disorder (Sha et al. 2022), obsessive–compulsive disorder (Kong et al. 2020), and schizophrenia (Gutman et al. 2022). These large-scale analyses impressively show that altered asymmetries are a robust finding in these disorders.

Hemispheric asymmetries are reflected in lateralized behavior such as handedness (e.g., hand preference or hand skill) (Ocklenburg and Gunturkun 2017). In the general population, around 10.6% of individuals show a left-hand preference, 9.3% a mixed-hand preference, and 18.1% demonstrate a preference for non-right-handedness (Papadatou-Pastou et al. 2020). Interestingly, the prevalence of left- and mixed-handedness are considerably increased in several mental disorders such as attention deficit hyperactivity disorder (Nastou et al. 2022), autism spectrum disorders (Markou et al. 2017), posttraumatic stress disorder (Borawski et al. 2022) and schizophrenia (Hirnstein and Hugdahl 2014). However, for some disorders, rates of non-right-handedness do not differ from the general population, for example, for patients diagnosed with depression (Packheiser et al. 2021). For several other disorders, meta-analytical integration of different study results is still needed before drawing conclusions regarding rates of non-right-handedness.

Similar to humans, hand preference or skill can be observed in animals where it is called paw preference in animals with paws or more generally “limb preference” to summarize all forms of limbs including flippers, fins and wings (Ocklenburg et al. 2019; Manns et al. 2021). In rodents, a meta-analytical integration of existing studies revealed that in terms of paw preference, rodents show strong individual lateralization but at the population level, no side preference was found (Manns et al. 2021). Another form of lateralized behavior in rodents is turning behavior when, e.g., navigating through mazes (Schwarting and Borta 2005) or open spaces such as the open field test (Mundorf et al. 2021a, 2022). In rodents, a preference for turning to the right has been reported and confirmed (Schwarting and Borta 2005; Mundorf et al. 2021a).

Given the multitude of studies conducted in animal models of several mental conditions, surprisingly few have focused on hemispheric asymmetry and lateralized behaviors. However, studying the clinical neuroscience of lateralization holds great potential, and animal models are needed to bridge the current gaps in knowledge.

Thus, this review aims to summarize the current knowledge regarding atypical hemispheric asymmetries from animal models of mental disorders and aims to unravel potential mental health consequences. To this end, the scientific database PubMed was searched for articles published until November 2022 on hemispheric asymmetry, lateralization, and animal models for mental conditions. This resulted in 22 studies included in this review (see Table 1).

**Table 1:** Summary of included animal studies grouped according to the disorder they aim to model

Animal model	Method/paradigm	Age + species	Hemispheric asymmetry	Behavior	Reference
Animal model of anxiety and fear	Fear conditioning	Male adult Sprague Dawley rats	↑ PKC βII levels in R amygdala	↑ Freezing	Orman and Stewart 2007
	Tail pinching/predator odor	Adult Long Evans rats	Males: ↑ dopamine in R amygdala + infralimbic cortex Females: ↑ dopamine in L amygdala	–	Sullivan et al. 2009
	Unilateral dopamine depletion in the ventromedial PFC	Male adult and female Sprague Dawley rats	L-sided lesions	Both: ↑ burying + ↑ sucrose consumption Females: ↓ anxiety in EPM;	Sullivan et al. 2014
			R-sided lesions	Males: ↑ anxiety in EPM	
Animal models of anhedonia and despair	Chronic mild stress	Male adult Wistar rats	↓ *Bdnf* and *Ntrk-3* in R frontotemporal cortex	↑ Anhedonia	Farhang et al. 2014
	Forced swim test	Female adult Wistar rats	–	L-sided head-turning: ↑ time immobile	Soyman et al. 2018
	Forced swim test	Male adult Wistar rats	–	L-sided paw: ↑ time immobile + ↑ reference memory ↔ Spatial memory	Ecevitoglu et al. 2020
Animal models of addiction/substance misuse	Ethanol containing diet, induced withdrawal	Male adult Long Evans rats	↑ Homovanillic acid ↓ dopamine + serotonin in mPFC ↓ Serotonin in amygdala (greater in R for R-preference rats)	Right-turning rat: ↑ ethanol drinking	Carlson and Drew Stevens 2006
	Nicotine injection	Adolescent + adult Sprague Dawley rats	Adolescents: ↓ asymmetry in R amygdala Adults: ↑ dendritic length in R amygdala + R infralimbic cortex	–	Bergstrom et al. 2010
Animal models of schizophrenia	Nogo-A-deficiency	Adult Sprague Dawley rats	Young adults: ↑ iNOS in L frontal + parietal cortices Old adults: bilateral ↓ NR1	–	Krištofiková et al. 2013
Animal models of neurodegenerative disorders	Transgenic Alzheimer’s model + isolation	Adult male 3xTg-AD mice	Atrophy of R hippocampus	Behavioral impairments	Muntsant and Giménez-Llort 2020
	5 amyloid models for Alzheimer’s	Adult mice	Asymmetric fibrillar plaque distribution	–	Sacher et al. 2020
	App^NL−G−F^ mouse model for Alzheimer’s	Adult mice	↑ TSPO in R amygdala	↑ Spatial memory	Biechele et al. 2021
	Unilateral AAV1/2-A53T α-synuclein injection in right SN	Male adult C57BL/6 mice	–	↑ Right paw use	Ip et al. 2017
	Unilateral 6-hydroxydopamine injection in right striatum	Adult C57/BL6J mice	–	↑ Right paw use	Mendes-Pinheiro et al. 2021
	Unilateral 6-hydroxydopamine injection in SN	Male adult C57BL/6 J mice	–	↓ Step length contralateral to injection	Broom et al. 2019
Animal models of stress exposure	Prenatal stress	Adult Sprague Dawley rats	↑ Alpha4 beta2 nAChR in R + L hippocampus; Non-stressed females: ↑ α7* nAChRs in L CA1	–	Schulz et al. 2013
	Maternal immune activation	Adolescent + adult Sprague Dawley rats	↓ Dopamine D2 receptor in bilateral mPFC	Adolescents: ↑ R turning; Adults: ↔ R + L turning	Mundorf et al. 2021a
	Maternal separation + social isolation	Adolescent + adult Sprague Dawley rats	–	↑ L turning bias	Mundorf et al. 2021b
	Limited bedding	Young Sprague Dawley rats after weaning	Males: ↑ perineuronal net density + synaptic plasticity in R amygdala	–	Guadagno et al. 2020
	Chronic social stress	Male adult Wistar rats	↑ Cytogenesis in R mPFC	–	Czéh et al. 2007
	Chronic restraint stress	Male adult Sprague Dawley rats	Stress eliminated hemispheric difference in infralimbic & prelimbic cortex Stress: ↓ apical dendritic length in left anterior cingulate cortex	–	Perez-Cruz et al. 2007
	Chronic restraint stress + Drug treatment	Male adult mice from B6C3H background	–	↑ L turns after stress Lithium treatment: ↑ absolute LQ	Mundorf et al. 2022

Arrows indicate no difference ( ↔), increased (↑), or decreased (↓) expression, levels or behavior compared to controls. If not further sp*ecified, both sex were included*

*R* right, *L* left, *mPFC* medial prefrontal cortex, *SN* substantia nigra

## Animal models for anxiety and fear and atypical asymmetries

The global prevalence of anxiety disorders is the highest of all mental disorders with 301·4 million estimated cases from a total of 654·8 million estimated mental disorder cases in 2019 (GBD 2019 Mental Disorders Collaborators 2022). Studies on structural asymmetries in patients diagnosed with anxiety disorders report mixed results, with some studies showing e.g., no atypical asymmetries in social anxiety (Bas-Hoogendam et al. 2017). A voxel-based meta-analysis in patients with social anxiety demonstrated that patients showed larger grey matter volume in the left precuneus, right middle occipital gyrus and supplementary motor area, but also smaller grey matter volume in the left putamen compared to healthy controls (Wang et al. 2018b). Importantly, the amygdala, an important structure for emotion processing, is highly lateralized (Ocklenburg et al. 2022) thus rendering the study of atypical asymmetries in anxiety a promising research objective.

In rodents, several anxiety-inducing models exist but only a few have focused on asymmetry. In a fear conditioning paradigm, adult male Sprague Dawley rats were conditioned to tones as cues and foot shocks, and neuronal PKC βII levels were measured in the lateral and basolateral amygdala. To avoid conditioning in the control group, animals were separately presented with tones or foot shocks, or neither. PKC βII levels were greater in the left amygdala when animals received neither shocks nor tons and when receiving pairs of tones and shocks. PKC βII levels were greater in the right amygdala in rats that were randomly presented with tones and shocks (Orman and Stewart 2007). Additionally, a subsample was tested for freezing behavior after conditioning. This analysis showed that more time spent freezing was predictive of greater increases in right-hemispheric PKC βII levels (Orman and Stewart 2007).

Another study exposed adult Long Evans rats to tail pinching or predator odor (fox urine) to induce stress and anxiety in the rats. Both exposures resulted in a greater dopamine release in the right basolateral amygdala and infralimbic cortex in males. However, females showed greater dopamine release in the left hemisphere (Sullivan et al. 2009).

The effects of a left vs. right unilateral dopamine depletion in the ventromedial prefrontal cortex on anxiety and aversion behavior were examined in a follow up study (Sullivan et al. 2014). To this end adult male and female, Sprague Dawley rats received unilateral 6-hydroxydopamine or vehicle injections. Only lesions in the left hemisphere resulted in changes in predator odor burying behavior with decreased latency to start burying and increased burying behavior in males and females. Similar effects were seen for the sucrose consumption with taste aversion where only the left-sided lesion resulted in reduced sucrose consumption in both sexes. However, the results from the elevated plus maze test reveal sex- and hemisphere-specific effects. Here, only right lesions reduced the open arm exploration time in males, while left lesions resulted in increased time spent in the open in females when compared to same-sex controls. The risk assessment in the elevated plus maze, defined as the number of stretches/attend postures, was affected by right lesions in opposite directions in males and females, with females demonstrating more such postures and males less (Sullivan et al. 2014).

To sum up, in rats, increased anxiety is associated with a greater right hemispheric activation in the amygdala. Additionally, left-sided dopamine depletion in the ventromedial prefrontal cortex seems to result in increased aversion while opposing results in anxiety are shown in the elevated plus maze test. Future studies should focus on other brain regions and their potentially asymmetric involvement in anxiety.

## Animal models of anhedonia and despair and atypical asymmetries

Self-perception and emotional processing show pronounced hemispheric asymmetries (Ocklenburg et al. 2014). Since these disruptions in these processes have been associated with depression, hemispheric asymmetries have been widely investigated in patients diagnosed with depressive disorders (Mundorf et al. 2021b). Indeed, several studies report altered functional asymmetry in depression (de Aguiar Neto and Rosa 2019; Mundorf and Ocklenburg 2021). However, a recently conducted ENIGMA consortium analysis found no alterations in structural hemispheric asymmetry in depression compared to the healthy population (de Kovel et al. 2019a). Therefore, animal models are crucial to understand the reasons for this conflicting results in the literature.

In animal models, symptoms of depression, such as behavioral despair or anhedonic behavior, are induced or assessed. A study with adult male Wistar rats applied a chronic mild stress protocol to induce depressive-like behavior. The resulting anhedonic behavior was assessed via the sucrose consumption test. When analyzing hemispheric mRNA expression in the frontotemporal cortex, anhedonic rats had lower levels of *Bdnf* and *Ntrk-3* in the right hemisphere compared to stress-resilient rats (Farhang et al. 2014). This is especially interesting given that the *Bdnf* gene, coding for a neurotrophic factor and *Ntrk-3*, a key gene in neurotrophic signaling, both play an important role in synaptic plasticity. Reduced synaptic plasticity has been reported in patients diagnosed with depression and in association with stress exposure is possibly leading to dysfunction of circuitries essential for mood regulation and cognitive function (Duman et al. 2016; Rahmani et al. 2022).

Another study assessed head-turning asymmetry in adult female Wistar rats to predict behavioral despair. Here, rats were water-deprived and forced to turn their head to access a water dispenser. Behavioral despair was induced with the forced swim test on the first day and measured on the second day followed by observing head-turning side preferences again. Indeed, rats showing a left head-turning bias demonstrated increased time spent immobile (thus more behavioral despair) in the second session while right-biased rats did not differ between sessions (Soyman et al. 2018).

Other groups have analyzed whether the paw preference of male Wistar rats is predictive of behavioral despair and spatial reference memory. First, rats were grouped as right or left-pawed using the paw preference test in which the animal has to reach through a narrow tube (only fitting one paw) to get the food reward. Then, the animals were tested in the forced swim test and the Morris water maze. Results show that rats with a left-sided paw preference demonstrated more behavioral despair but also had better reference memory than right-pawed rats while spatial learning performances did not differ between groups (Ecevitoglu et al. 2020).

To conclude, motor asymmetry seems to be a robust marker for behavioral despair and anhedonic behavior. Studies revealed that higher gene expression levels in the right hemisphere are associated with increased anhedonic behavior, whereas left-sided asymmetry in head-turning and paw preference is associated with increased behavioral despair.

## Animal models of addiction/substance misuse and atypical asymmetries

Hemispheric asymmetries have not been the focus of most studies including patients diagnosed with substance-related and addictive disorders. But recently, the ENIGMA Addiction Working Group performed a mega-analysis comparing cortical and subcortical asymmetries between patients addicted to either nicotine, cannabis, alcohol, methamphetamine, or cocaine and non-addicted controls. Indeed, patients addicted to one of the substances demonstrated a reduced volume of the right nucleus accumbens, a key region of the reward circuit (Cao et al. 2021). However, animal studies including hemispheric asymmetries have so far focused on the amygdala and the prefrontal cortex.

In animals, the consequences of addiction or substance misuse are often studied by repeatedly exposing the animal to or injecting the substance. One group studied the effect of ethanol consumption and following withdrawal on asymmetric dopamine, serotonin, norepinephrine, and metabolite concentrations in the amygdala and the medial prefrontal cortex. Therefore, adult male Long Evans rats were first classified as right-turning, left-turning, or as having no turning side preference and then fed with a liquid ethanol-containing diet over 2 weeks versus a control diet. Neurotransmitter levels were assessed in the acute withdrawal phase. The results show that rats subjected to an ethanol-containing diet had lower levels of dopamine and serotonin in the medial prefrontal cortex. Furthermore, the ratio between the metabolite homovanillic acid and the neurotransmitter dopamine was increased in the medial prefrontal cortex. In the amygdala, only serotonin levels were lower compared to controls. Interestingly, rats with an intrinsic right-turning preference demonstrated greater changes in the right hemisphere (Carlson and Drew Stevens 2006). Animal and human studies suggest that dopamine and serotonin play a crucial role in mood, reward processing and prediction. Moreover, they demonstrated that the corresponding receptors are targeted by drugs of abuse. These facts make dopamine and serotonin interesting targets in addiction research (Kirby et al. 2011; Fischer and Ullsperger 2017).

In a follow-up experiment, rats were again fed with the ethanol-containing liquid diet over 2 weeks and then tested for ethanol versus sucrose or water consumption. Here, rats with a right-turning preference drank noticeably more ethanol than rats with a left-turning preference but not more sucrose water. Additionally, rats with right-turning bias drank more ethanol following a withdrawal period (Carlson and Drew Stevens 2006).

To investigate the effect of nicotine exposure on neuron morphology asymmetries in the amygdala and infralimbic cortex, Sprague Dawley rats were injected six times with nicotine or saline during adolescence or adulthood. Exposure in adolescence did not lead to changes in dendritic morphology 20 days after nicotine exposure. However, the control group showed increased dendritic length in the right amygdala while this hemispheric asymmetry was not present in nicotine exposed rats. Nicotine exposure during adulthood resulted in right hemispheric increased dendritic length (Bergstrom et al. 2010).

In sum, animal models hint towards a right-turning preference leading to increased ethanol consumption after repeated exposure to ethanol. Furthermore, they show a greater decrease in neurotransmitter levels in the right hemisphere. Neuronal consequences of nicotine exposure seem to be more pronounced in the right than in the left hemisphere. Of note, it is unclear to what extent models forcing the animal to an exposure represent the human condition. Future studies integrating behavioral asymmetries and asymmetric neurotransmitter distribution that include structures such as the nucleus accumbens are needed to disentangle altered asymmetries in addiction.

## Animal models of schizophrenia and atypical asymmetries

One of the most frequently studied mental disorders in clinical lateralization research is schizophrenia (Mundorf et al. 2021b). A recently conducted large-scale study from the ENIGMA consortium identified changes in shape asymmetry across several regions in patients diagnosed with schizophrenia compared to the healthy population (Gutman et al. 2022).

Studies in animal models of schizophrenia and atypical asymmetries are rare. In a Nogo-A-deficient rat model for schizophrenia, expression asymmetries in the cortical NMDA receptor-nitric oxide synthase pathway were analyzed in young and old adult Sprague Dawley rats and controls. In younger adult Nogo-A-deficient rats, increased inducible nitric oxide synthase (iNOS) activity, that is essential in inflammatory processes and associated with oxidative stress, was shown in the left hemisphere of frontal and parietal cortices compared to age-matched controls. Older Nogo-A-deficient rats demonstrated a bilateral decrease in the expression of the NMDA receptor subunit NR1 and increased expression of the NMDA receptor subunit NR2B in the right hemisphere. At both ages, changes in interactions within the investigated pathway were observed in the experimental groups. The authors conclude that young Nogo-A-deficient rats experience accelerated aging and abnormal frontoparietal cortical interactions similar to findings in humans (Krištofiková et al. 2013). The increased iNOS activity is in line with the hypothesis of increased pro-inflammatory activity in schizophrenia (Esshili et al. 2020; Fond et al. 2020). Moreover, alterations in the cortical NMDA receptor-nitric oxide synthase pathway as well as changes in NMDA receptor subunit NR1 and NR2B expression have been linked to schizophrenia and altered lateralization (Clinton and Meador-Woodruff 2004; Nudmamud-Thanoi and Reynolds 2004; Li and He 2007; Ocklenburg et al. 2011).

## Animal models of neurodegenerative disorders and atypical asymmetries

Neurodegenerative disorders have a special link to hemispheric asymmetries as the most important finding is the role of lesion laterality. This is especially the case for Parkinson’s disease, in which disease severity and experienced symptoms are influenced by the hemisphere affected (Chen et al. 2021; Lubben et al. 2021; Mundorf and Ocklenburg 2021; Steinbach et al. 2021). Similarly, asymmetric atrophy is reported in patients suffering from Alzheimer’s disease, and that altered asymmetries are associated with worse symptoms (Low et al. 2019).

In animals, several transgenic and genetically modified mouse and rat models exist to depict alterations associated with Alzheimer’s disease (Drummond and Wisniewski 2017). To study the different facets of Parkinson’s disease, transgenic or viral vector-mediated models based on genes linked to the disease are used or animals are unilateral exposed to neurotoxins that induce neuronal dopamine depletion and motor impairments (Konnova and Swanberg 2018). Given that the unilateral lesion model is widely established to induce asymmetric motor impairments, listing all relevant studies is beyond the scope of this review. To clarify the mechanism of action, only a few recent studies will be included.

To model the advanced stages of Alzheimer's disease and the impact of long-term isolation, male transgenic 3xTg-AD mice, a triple-transgenic model (3xTg-AD) where mice from a C57BL/6 genetic background harbor PS1(M146V), APP(Swe), and tau(P301L) transgenes, were socially isolated and compared to non-transgenic mice as well as non-isolated transgenic mice (controls). Mice from this transgenic line progressively develop plaques and tangles as well as synaptic dysfunction all of which is present in Alzheimer’s disease (Oddo et al. 2003). Along with behavioral changes similar to the clinical picture of Alzheimer’s disease, asymmetric atrophy of the right hippocampus was found in the 3xTg-AD mice that was reinforced by isolation (Muntsant and Giménez-Llort 2020).

Another study analyzed asymmetries of fibrillar plaque burden in five frequently investigated amyloid mouse models for Alzheimer’s disease. The team examined hemispheric asymmetries of amyloid-β and 136 18-kDa translocator protein (TSPO; a marker of microglia activation) via cross-sectional PET scans and observed that over 30% of the experimental animals had strong asymmetric fibrillar plaque distribution but no hemispheric predominance across models was found. In terms of microglia activation, asymmetric amyloid-β accumulation was positively correlated with ipsilateral TSPO expression (Sacher et al. 2020).

In a follow-up study, the research team wanted to further characterize hemispheric microglia activation in the App^NL−G−F^ mouse model for Alzheimer’s disease. Therefore, female and male mice underwent cross-sectional TSPO-PET and β-amyloid-PET scans and were tested in the Morris water maze for spatial memory performance afterward. Right lateralized TSPO expression in the amygdala predicted better spatial memory with no effect of amyloid-β or left-lateralization on spatial memory (Biechele et al. 2021).

Animal models for symptoms of Parkinson’s disease often induce an unilateral lesion, e.g., by injecting 6-hydroxydopamine, in the right nigrostriatal pathway resulting in motor impairments similar to changes seen in patients with Parkinson’s disease (Ip et al. 2017; Mendes-Pinheiro et al. 2021; Sun et al. 2022). Motor asymmetry is often assessed by examining the limb use after unilateral lesion resulting in asymmetric use of the ipsilateral paw (Ip et al. 2017; Mendes-Pinheiro et al. 2021). Others also found asymmetries in temporal and spatial walking metrics such as step length asymmetry in line with findings in human subjects with Parkinson’s disease (Broom et al. 2019).

In conclusion, animal models of Alzheimer’s disease indicate an asymmetrical accumulation of fibrillar plaque burden as well as an asymmetrical atrophy predominantly associated with the right hemisphere. Mouse and rat models of Parkinson’s disease induce symptoms via right-sided lesions.

## Animal models of stress exposure and atypical asymmetries

Stress exposure is a risk factor to develop psychopathologies, especially early exposure can result in long-lasting neurobiological alterations (Lupien et al. 2009; Kumsta et al. 2017; Abraham et al. 2022). Interestingly, several early life factors as well as stress exposure are associated with an increased rate of left-handedness and atypical hemispheric asymmetries (de Kovel et al. 2019b; Berretz et al. 2020; Mundorf et al. 2020). Of note, besides non-human mammals and humans, the predominantly greater right hemispheric involvement after stress has also been reported in fish, reptiles and birds (Ocklenburg et al. 2016).

Animal models of stress exposure use different developmental windows, such as prenatal, early postnatal, or during adulthood, to subject the animal to a single stressor or chronic stress. Nowadays, researchers have established a variety of stressors to mimic different aspects of stress exposure and have even established genetic models to be more susceptible to stress (Atrooz et al. 2021). This paragraph will list studies chronologically according to the time of stress exposure starting with prenatal exposure until stress in adulthood.

To investigate the effects of prenatal stress on hippocampal nicotinic acetylcholine receptor (nAChR) expression, pregnant Sprague Dawley rats were subjected to unpredictable variable stressors two to three times daily during the last week of gestation. The offspring was group-housed until adulthood. Then the expression of subunits of nAChR was analyzed using autoradiography. Studying the distribution of nAChR expression is an interesting target as nAChR are involved in fast synaptic transmission and in cognitive processes such as learning and memory (Hogg et al. 2003). Given their role in memory and learning analyzing their hippocampal expression may render important information on long-lasting impairments. Moreover, animal studies revealed that pre-synaptic alpha3 or alpha4 nAChR subunits in combination with the beta2 subunit may mediate dopaminergic and noradrenergic release, whereas the alpha7 subunit may control the release of glutamate (Hogg et al. 2003). Of interest, recent neurochemical and pharmacological studies reinforce that mainly the subunits alpha4 beta2 and alpha7 are involved in psychiatric disorders (Araki et al. 2002). Prenatally stressed adult males and females had higher alpha4 beta2 nAChR levels spanning the whole hippocampus. Moreover, non-stressed females had left-sided lateralization of alpha7 nAChRs in the CA1, compared to stressed females. Changes in the distribution of nAChR subunits may affect the neurotransmitter release in respect to their properties. The authors thus propose that prenatal stress exposure alters the developmentally normal lateralization of alpha7 nAChRs which may render the individual more vulnerable to the development of mental disorders (Schulz et al. 2013).

Another study analyzed turning behavior in a maternal immune activation model. Therefore, pregnant Sprague Dawley rats were injected with polyinosinic:polycytidylic (PolyI:C) at gestational day 15 to induce a maternal immune reaction. Adolescent and adult offspring were then analyzed for turning behavior examined in the open-field test. In adolescents, maternal exposure resulted in a rightward turning bias compared to controls whereas, in adults, maternal immune activation led to an absence of turning bias that may be associated with reduced asymmetry. The authors furthermore analyzed dopamine D2 receptor expression in the prefrontal cortex and found bilateral reduced expression after stress in adolescence (Mundorf et al. 2021a). The prefrontal cortex develops rather late during gestation, e.g., corticogenesis for the dorsolateral prefrontal cortex starts in the 8th gestational week in humans (Selemon and Zecevic 2015). Thus, it is vulnerable during gestation where disruptions can lead to dysfunction associated with schizophrenia-like symptoms (Selemon and Zecevic 2015). The neurotransmitter dopamine is hypothesized to mediate between stress exposure and schizophrenia-like pathology (Selemon and Zecevic 2015). This involvement of dopamine in schizophrenia is further underlined given that the dopamine D2 receptor is the major target for antipsychotic medication (Wang et al. 2018a). Thus, changes in dopamine D2 receptor expression after a maternal immune challenge may render the individual vulnerable to develop a schizophrenia-like pathology.

Studies focusing on early life stress usually subject the pups in their early postnatal days to environmental changes such as limited bedding or separation from the dam (Guadagno et al. 2020; Mundorf et al. 2020). In a study separating the pups from the litter and the dam (called maternal separation), the effects of early separation on turning behavior were assessed. Therefore, pups were separated from postnatal days 2–20 for 4 h daily from their litter and dam. A subsample was additionally exposed to social isolation after weaning while the littermates were group-housed. Turning behavior was then measured while navigating through the elevated plus maze. Cumulative exposure to stress resulted in a strong left-turning bias compared to controls independent of sex and age (Mundorf et al. 2020).

A different study focused on the consequence of early stress by exposing the pups to limited bedding from postnatal days 1–10 in female and male Sprague Dawley rats. After weaning on postnatal day 22, neuronal plasticity in the amygdala was measured. Here, exposure to limited bedding led to increased perineuronal net density and synaptic plasticity in the right amygdala of males but not females (Guadagno et al. 2020).

Concerning the consequences of chronic stress exposure in adulthood, a study exposed adult male Wistar rats to 5 weeks of daily chronic social stress with the resident–intruder paradigm. Additionally, some animals were treated orally with fluoxetine over the last 4 weeks, an antidepressant that inhibits the extracellular reuptake of serotonin into the presynaptic cell resulting in a repeated stimulation by serotonin of receptors of the recipient cell which leads to an increase in synaptic signaling. Then cell proliferation in the left and right medial prefrontal cortex was analyzed and compared to controls. Stress exposure resulted in increased cytogenesis in the right medial prefrontal cortex whereas cytogenesis rates were higher in the left hemisphere in controls. Treating the animals with fluoxetine abolished the found hemispheric asymmetries in both conditions (Czéh et al. 2007).

Others examined the consequences of chronic restraint stress on pyramidal neurons dendrite morphology in the prefrontal cortex. To this end, male adult Sprague Dawley rats were restraint over 6 h for 21 days and neuronal morphology analyzed. In control animals, hemispheric differences were obvious with longer apical and distal dendrites in the right prelimbic cortex and longer proximal apical dendrites in right infralimbic cortex compared to the left hemisphere. In stress-exposed animals, both hemispheric differences were abolished. In the anterior cingulate cortex, no hemispheric difference was visible in controls, but stressed rats demonstrated reduced apical dendritic length in left anterior cingulate cortex. The authors conclude that stress results in predominantly selective effects on the right hemisphere (Perez-Cruz et al. 2007).

One study investigated the effects of chronic stress exposure in adult mice on turning behavior (Mundorf et al. 2022). Here, male mice from a B6C3H background were analyzed. More precisely, a glutamine synthetase reporter mouse model (B6C3H-Glultm^(T2A-LacZ-loxP-T2A-Tk-1-FRT-loxP-T2A-Fluc-FRT)Arte^) with a genetical B6C3H background was studied. In a previous study including this mouse model it was shown that lithium treatment prevented stress-induced increased glutamine synthetase activity in the hippocampus (Mundorf et al. 2019). In this follow up study by Mundorf et al. (2022), mice were injected with either ketamine hydrochloride, lithium carbonate, or sham (sodium chloride) followed by 2 h of chronic restraint stress over seven days. As therapeutic drugs in mental health, ketamine hydrochloride is administered as a fast-acting antidepressant with mood lifting properties (Smith-Apeldoorn et al. 2022) whereas lithium carbonate is used as mood stabilizer, mostly to treat bipolar disorder and suicidal behavior (Cipriani et al. 2013; Volkmann et al. 2020). Turning behavior was assessed 2 days after the last restraining but ongoing treatment. Therefore, side preferences when turning in the open field and novel object test were measured. Mice that were exposed to stress and saline injection showed more left than right turns in the novel object test meanwhile mice treated with ketamine or lithium turned equally often to the left and right side. When analyzing the absolute lateralization quotients (indicating the strength of turning bias), mice treated with lithium carbonate demonstrated a noticeably stronger bias compared to ketamine hydrochloride treated mice but only in the novel object test (Mundorf et al. 2022).

Summing up, studies highlight that exposure during early periods as well as in adulthood leads to behavioral left-sided preference or attenuated asymmetry behavior similar to findings in patients. On the neuronal level, prenatal stress, and exposure in adulthood results in increased right-sided activity. Interestingly, treating the animals with psychiatric drugs seems to reverse stress-induced asymmetric alterations.

## Discussion

The results of animal studies modeling symptoms of mental disorders suggest an association between psychopathological symptoms and an altered side preference along with atypically asymmetric increased neuronal activity. In detail, increased anxiety and anhedonic behavior are linked to increased right-hemispheric neuronal activity and a left-sided behavioral bias is associated with increased behavioral despair. Similarly, stress exposure leads to atypical left-side preferences in behavior as well as increased right-sided neuronal activity. In contrast, addiction behavior as a result of substance exposure is associated with increased consumption in right-bias animals and a greater decrease in neurotransmitter levels in the right hemisphere. Transgenic mice models of Alzheimer’s disease indicate an asymmetrical accumulation of fibrillar plaque burden as well as an asymmetrical atrophy predominantly associated with the right hemisphere. Models of Parkinson’s disease apply unilateral lesion to the right hemisphere. Across studies, two crucial factors influencing atypical asymmetries arose independently of disorder modeled: sex and developmental age with partially opposing effects found in females and males as well as in adolescents versus adults (Sullivan et al. 2009, 2014; Bergstrom et al. 2010; Krištofiková et al. 2013; Schulz et al. 2013; Mundorf et al. 2021a). However, around half of the studies did only include male animals and thus, one cannot conclude general sex-dependent alterations in hemispheric asymmetries.

Human and animal studies have repeatedly shown that stress exposure results in stronger right-hemispheric activation that is connected to increased leftward behavioral asymmetry (Czéh et al. 2007; Perez-Cruz et al. 2007; Farhang et al. 2014; Ocklenburg et al. 2016, 2022; Guadagno et al. 2020). Given that most models presented in this review apply a stress-based paradigm to induce symptoms of e.g., fear, anxiety, despair, and anhedonia, it is difficult to disentangle whether the atypical asymmetries can be allocated to the specific disorders or rather to stress as a cause. Especially, as the models without explicit stress exposure, namely the transgenic Alzheimer’s models as well as the models of addiction are not associated with increased left-sided behavior but rather with a right-sided preference for the latter. In the patient context, three different types of associations have been proposed between atypical asymmetries and psychopathology: non-specific, diagnosis-specific, and symptom-specific association (Mundorf et al. 2021b). Assuming the hypothesis of stress-induced altered asymmetries, we propose a fourth possible association: the cause-specific association. It is yet to be uncovered which one may hold true or whether several associations lead to atypical asymmetries.

Interestingly, early exposure resulted in similar alterations as exposure in adults with adolescence and early adulthood marking a time of great neuronal change (Galván 2014, 2017) that could lead to the found incoherent alteration in asymmetry throughout development. Given the small number of studies that included adolescence, no final conclusion can be drawn yet. More studies are needed that include several developmental stages, especially in light of increasingly earlier ages of onset for mental disorders (Solmi et al. 2021).

Another interesting finding is the predictive value of left-lateralized behavior for behavioral despair in animals. In particular, as studies on human handedness do not find an increased ratio of left- or mixed-handedness in patients with depression (Packheiser et al. 2021). Results from animal studies may thus point to associations between asymmetries and more subtle depressive symptoms instead of the clinical picture of depression. Possibly, subclinical symptoms, such as despair, are more related to stress-induced alterations than to alterations associated with depression. Also, microstructural asymmetries might be more affected than structural asymmetries (Mundorf et al. 2021c). However, given the current knowledge, this remains highly speculative.

Besides the direction of lateralization, e.g., the preferred side, analyzing the strength of lateralization may render important insights. Especially given that, for some behaviors such as paw preference, meta-analytical integration demonstrated that rodents show strong individual lateralization but no population-wide side preference (Manns et al. 2021). Thus, analyzing whether animals of one group show generally strong side preferences independent of side versus no side preferences can be useful (for example Mundorf et al. 2022).

Lastly, the two studies on chronic stress exposure in adulthood reveal an interesting finding on the effects of psychopharmacological treatment on hemispheric asymmetries: treatment with the antidepressants fluoxetine and ketamine seems to abolish stress-induced atypical asymmetries whereas lithium, a mood stabilizer, increased general asymmetry behavior independent of side (Czéh et al. 2007; Mundorf et al. 2022). Of course, more research is needed to further uncover the effects of drug treatment on hemispheric asymmetries.

The study of hemispheric and behavioral asymmetries currently holds some limitations. First, it is difficult to objectively assess and score lateralized behavior as most behaviors are still being assessed manually. Here, new computational methods are needed that allow for an objective and comparable scoring of behaviors. Second, most of the included studies only focused on regions of interest or one specific behavior. This is understandable as whole-brain analyses, and several behavioral tests are quite expensive and laborious. However, the current studies are biased towards changes in other brain regions or might miss behavioral asymmetries. Third, studies often do not distinguish between sub-regions when analyzing neuronal changes. But nuclei-specific lateralization is already known, at least for some regions such as the amygdala (Ocklenburg et al. 2022). Consequently, when investigating hemispheric gene or protein expression, studies need to disentangle nuclei-specific changes in order to truly detect atypical asymmetric expression patterns. Forth, little is known about sex-specific and age-dependent effects on hemispheric asymmetries in animal models of mental disorders. More research is needed to place the current findings.

## Conclusion

In conclusion, animal models of mental disorders demonstrate atypical hemispheric asymmetries similar to findings in patients. Particularly, increased left-sided behavior and greater right-hemispheric activity was found across models applying a stress-based paradigm. However, sex- and age-dependent effects on atypical hemispheric asymmetries are present that require further investigation. Animal models enable to analyze hemispheric changes on the molecular level which may be most effective to detect early alterations.

## Data Availability

Data sharing not applicable to this article as no datasets were generated or analysed during the current study.

## Abbreviations

nAChR: Nicotinic acetylcholine receptor
PolyI:C: Polyinosinic:polycytidylic
TSPO: 136 18-KDa translocator protein
